# Fostering needs assessment and access to medical rehabilitation for patients with chronic disease and endangered work ability: protocol of a multilevel evaluation on the effectiveness and efficacy of a CME intervention for general practitioners

**DOI:** 10.1186/s12995-017-0168-3

**Published:** 2017-08-04

**Authors:** Stephan Fuchs, Katrin Parthier, Andreas Wienke, Wilfried Mau, Andreas Klement

**Affiliations:** 1 Institut für Allgemeinmedizin, Martin-Luther-Universität Halle-Wittenberg, Magdeburger Straße 8, 06112 Halle (Saale), Germany; 2 Institut für Rehabilitationsmedizin, Martin-Luther-Universität Halle-Wittenberg, Magdeburger Straße 8, 06112 Halle (Saale), Germany; 3 Institut für Medizinische Epidemiologie, Biometrie und Informatik, Martin-Luther-Universität Halle-Wittenberg, Magdeburger Straße 8, 06112 Halle (Saale), Germany

**Keywords:** Medical rehabilitation, Need detection, General practitioner, Continuing medical education, Work ability

## Abstract

**Background:**

Studies show that endangered work ability (EWA) can be maintained or restored through medical rehabilitation (MR). For patients, general practitioners (GP) represent an important point of access to MR in outpatient care. However, many different barriers and shortcomings hinder GPs in both timely detection of the need for MR and the recognition of its potentials for their EWA-patients. These are necessary if GPs are to adequately inform patients about MR options and successfully support applications for MR. This study describes the evaluation of a continuing medical education (CME) module designed to improve rehabilitation-related practical performance of GPs regarding a) subjective satisfaction of GPs with the CME module, b) stability of attitudes and knowledge over time regarding rehabilitation, and c) subjective and objective changes in MR-related competencies needed to support MR applications.

**Methods:**

This study is an open, non-randomised, pre-post-intervention study. The intervention involves a CME module for GPs (*n* = 1365) in the German state of Saxony-Anhalt on the topic of medical rehabilitation in connection with the federal German pension fund (Deutsche Rentenversicherung). The module will be initially held as regularly scheduled meetings in moderated GP quality circles (QC), and then offered as a written self-study unit. At the end it will be evaluated by the GPs. The study’s primary focus is on the organizational practice as measured by the number of approved MR applications supported by medical reports submitted by the participating GPs in the 6 months before and 6 months after the CME module. Other study aims involve measuring self-perceived competencies of GPs, as well as their attitudes towards and knowledge of rehabilitation (both upon completing the CME and 6 months later). In addition, the level of satisfaction with the CME module will be analysed among participating GPs and QC moderators (as CME facilitators).

**Discussion:**

Implementing targeted CME on complex topics such as those involving barriers is possible, even promising, when using QCs and their moderators. Of particular importance is how aware moderating physicians are of the relevance of MR need detection and access.

**Ethics and dissemination:**

The ethics committee of the Martin-Luther-Universität Halle-Wittenberg has registered this study under the number 2014–13. The study will be reported on in peer-reviewed journals and at national and international conferences. The results will be available to current and future initiatives aiming to improve detection of MR need and making MR accessible to EWEC patients needing such support to minimize the effects of chronic disease on their livess.

**Trial registration number:**

German Clinical Trials Register (ID number DRKS00006188) and WHO International Clinical Trials Registry Platform, Universal Trial Number (UTN) U1111–1158-8334.

## Strengths and limitations

- Our intervention is important for every participant (QC, postal) and his daily patient treatment
- This study investigates the effect of a CME module focusing on detection of EWA patient needs and access to MR in a pre-post comparison using a large cohort of GPs in the German federal state of Saxony-Anhalt.
- The evaluation will be done on different levels: subjective satisfaction of the GPs, stability of GP attitudes, knowledge and self-rated competencies regarding rehabilitation over time as well as the objective changes in CME-based competencies as measured in terms of approved MR applications for the participating GPs.
- We have a self-selection of participants into the two types of interventions, unfortunately we have not a double-blinded randomized study.
- The self-selection has a significantly influence on results of these interventions.
- The evaluated CME module uses both the innovative format of the moderated quality circle and the conventional medium of printed materials for independent study, allowing for subgroup analyses of the effectiveness of the two formats.
- With the accompanying risk of selection bias, recruitment of the GPs permits them to choose between taking the CME module in the QC setting or as independent study.
- For some participants, there are different starting dates for the pre-post evaluations due to varying meeting schedules for some QCs or as a result of independent study.

## Background

### Endangered work ability and medical rehabilitation

Partaking in gainful employment is essential to individual wellbeing, social involvement and quality of life. In contrast, chronic disease is a significant predictor of a limited ability to practice a profession and/or endangered work ability (EWA) with considerable disadvantages for the individual and society, [1]. Numerous studies demonstrate that EWA due to chronic disease can be maintained or restored through medical rehabilitation (MR) with the goal of returning to work, [2]. Common characteristics can be seen in examples of MR for reasons as diverse as lower back pain, inflammatory arthritis or depression, which are different in their somatic and psychological components: successful interventions restore the balance between individual abilities and workplace demands through changeable factors-either on the level of the individual’s abilities or the demands of the workplace, [3–5]. Medical rehabilitation is an important prevention instrument to inhibit individual increasing chronical diseases. For the individual patient, disease-specific limitations of abilities (e.g. problems concentrating) are, of course, many-sided often requiring complex intervention with many components, for instance patient training, physiotherapy, occupational therapy, psychoeducation and occupational counselling, etc., [2]. Within Germany’s complex administrative system, the most important insurance provider to cover the cost of MR in terms of numbers is the federal German pension fund: the Deutsche Rentenversicherung (DRV). Distinguishing itself through a wide range of in- and out-patient rehab facilities and programs, the DVR is ahead even of the statutory health insurance providers. The applicant for such services and resources is always the individual EWA patient, [6].

### The role of the general practitioner in accessing medical rehabilitation

According to Starfield, coordination of care with other medical care providers is one of the four pillars of primary care, with particular importance for chronically ill patients, [7]. Within this context general practitioners (GP) represent an important point of access to MR in the outpatient setting; primary care physicians are responsible for recognizing need, informing patients about MR programs, supporting applications for rehabilitative medical services across the many sectors of care with well-founded medical reports, and ensuring follow-up care after successful MR. In particular, the medical reports appended to the patient’s application for rehabilitation should contain the pertinent information for evaluation by the insurance provider (in this case the DRV). However, according to social medicine experts these reports often contain insufficient information, [8]. Each year approximately 1,700,000 applications for medical rehabilitation are submitted to the DVR, of these about 1,300,000 are approved. In the course of this, those participating in the process become aware of many barriers in patients, physicians, and the system that stand between an application and its approval, [8–15]:

### Barriers and shortcomings

In a series of surveys, practicing physicians reported substantial problems not only concerning information on detecting the need for MR, the necessary steps patients need to take to apply for MR, and the requirements that supporting medical reports need to fulfil, but also concerning the physicians’ own lack of clarity regarding the criteria for application approval and for asserting objections to rejected applications, [8]. Likewise, the flow of information between the healthcare providers, routine measures within medical practices and, especially, the integration of non-medical health professions into the detection of need for MR and the application process (to relieve the physician workload), all indicate a considerable potential for improvement, [9]. Within other healthcare systems and professional groups similar needs and barriers have also been identified that affect MR case management, [10]. For EWA patients it is the GP’s encouragement and support, alongside psychosocial and family-related factors, that significantly affect a patient’s intention to actively pursue and apply for MR, [11].

It is possible that the main challenge in recognizing the need for MR, and successfully applying for it, can be found in the doctor-patient relationship. Usually, the existence of a potential case of EWA becomes apparent to a GP after a series of episodes during which the patient is unfit for work and seeks a sick-leave certificate, [12]. Qualitative studies show that many practicing physicians see themselves as their patients’ “advocate” and issue the requested medical attestations without further questioning or intervention regarding EWA partly not to jeopardize the doctor-patient relationship, [12]. At the majority of medical practices depressive disorders, for instance, do not merit (pro)-active or EWA-focused approaches as long as other professionals (e.g. psychotherapists) or the patient does not insist on them, [13]. The willingness of GPs to change this situation is rather weak despite the availability of practice- and patient-centred support to relieve workloads, [14].

Patients, in turn, are frequently prevented from forming the intention to pursue and plan for MR because of the negative expectations of their family or social environments, lack of self-efficacy and a (perceived) lack of support, [11]. On top of this, the information about MR compiled specifically for patients is also difficult to understand, [15]. Implementing the strategic goal of pairing eligible patients with the right MR at the right point in time appears to be equally complex, [15].

### Interventions to foster continuing medical education

The number of sick days and incidences of early retirement due to EWA can be reduced by MR (just as by vocational rehabilitation or combinations of the two), [2]. Due to the great social and financial importance of EWA not only for the individuals affected, but also society as a whole, interventions to improve recognition of MR need and access are both imperative and promising, [1, 2].

Approaches to intervention that have been positively evaluated use continuing medical education (CME) to impart knowledge and change physicians’ attitudes, prevention and treatment strategies. There are three different methods to communicate CME content, each of which can be employed separately or in combination:

- Printed CME materials with little effect on process parameters and questionable effects on patient-centred outcomes and unclear clinical relevance, [16].
- Audit and feedback on CME with moderate to strong effects—including on patient-centred outcomes particularly if the intervention is repeated among medical colleagues in spoken and written form and covered using a clear agenda, [17].
- Meetings and workshops on CME can, alone and in combination with other intervention formats, have similar effects as seen with audits and feedback particularly if they focus on topics and outcomes that are considered relevant by the participants, [18].

These formats are used for MR-related CME interventions in current study protocols, frequently in combination or connection with other formats, [19, 20]. A special role is increasingly being played by patient-centred interventions, in which particular risk groups are proactively addressed and/or specially compiled information is presented along with instruments for self-screening, [21, 22].

### Quality circles for CME

Quality circles (QC) are understood to be regular meetings of professionals from the same occupation or interdisciplinary working groups with a defined group of participants, shared goals and professional approaches; leadership is usually taken on by a peer as moderator, [23]. Through moderated interaction with printed or audio-visual materials covering CME content, the QC embodies, in an easily accessible manner, both the format of the meeting/workshop and the elements of audit and feedback through the regularity of the meetings and direct inquiries about individual approaches, [23, 24]. In connection with Germany’s special system of compensation for providing primary care (*Hausarztzentrierte Versorgung,* abbreviated as HzV), QCs as a format for CME (mandatory for HzV physicians) have taken on great importance in recent years, [25, 26].

### Required research

Despite very promising initial evidence from surrogate parameters and secondary data, there is currently little evidence available that points to the effectiveness and efficacy of imparting concrete CME content using the QC format, [27–29].

Evidence is particularly lacking to suggest whether or not controversial topics associated with barriers, such as the MR-related CME content, can be effectively communicated using QC, [23, 27]. For this reason, we see a need, in this study, to evaluate a CME module in terms of improving the rehabilitation-related knowledge and competencies of GPs on the following levels:

- Subjective GP satisfaction with the CME module,
- Stability of MR-related attitudes and CME-related knowledge over time, and
- Subjective and objective changes in CME-related competencies needed to support MR applications.
- sensitizing for MR as an instrument for prevention

## Methods

### Aims

The primary aims of this study are to implement and evaluate a CME module for all GPs in Saxony-Anhalt focusing specifically on detecting EWA and facilitating access to MR. Data will also be drawn from defined time periods before and after the module.

### Trial design

This study is an open, non-randomised, pre-post-intervention study. Primary and secondary analyses of the data will be undertaken to evaluate the intervention.

Using secondary data from the DRV Mitteldeutschland, responsible for servicing Saxony-Anhalt, the periods of time 6 months before and 6 months after the intervention will be investigated to see how the approval rates change for MR applications made by insured patients for whom the participating GPs have submitted medical reports.

**Fig. 1:**
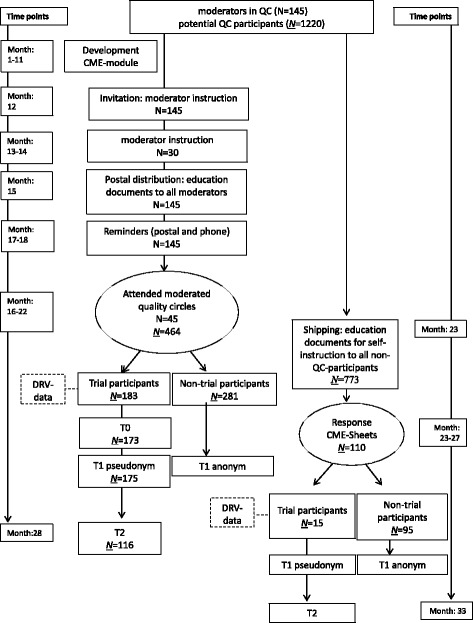
Trial design of our study: an open, non-randomised, pre-post-intervention study. The intervention (quality circle / postal) involves a CME module for GPs

Primary data collection will take place using questionnaires. The satisfaction of the participating physicians with the module will be assessed immediately after completing the continuing education (t1). The influence of the intervention on GP knowledge about and their attitudes towards rehabilitation will be assessed at two different points in time (t0 = immediately prior to the intervention; t2 = 6 months afterwards). The self-rated CME-related competences will be assessed after completing the continuing education (t1) and 6 months afterwards (t2) (Fig. 1).

### Participants & recruitment

General practitioners (i.e. specialists in General Practice, specialists in internal medicine working in Primary Care) participating in special forms of additional compensation schemes (HzV) in Saxony-Anhalt were recruited for this study.

Physicians participating in HzV are required to regularly attend QC as part of an ongoing obligation to pursue continuing education and professional training. This applies to 90% of all GPs in Saxony-Anhalt. At the start of the study, in Saxony-Anhalt 145 GPs served as QC moderators for 1220 GPs (HzV) as potential QC participants.

The initial recruiting phase relies on the QC moderators: all 145 moderators will be informed about the module and invited to an informational meeting on the module. Regardless of whether or not the moderators attend this informational meeting, materials on the module and its evaluation will be sent to all 145 moderators for use in their QCs. Both, the conduction of the module and participation in the study are voluntary for the moderators. Moderators who decide to implement the module will distribute pertinent information about this interventional study and the CME module to their QC participants and then conduct the module in the form of on-site meetings.

During the second recruiting phase, information on the study and continuing education module will be sent to all HzV physicians who have not participated in a rehabilitation-related QC to offer them the option of covering the material as an independent study unit. The physicians who elect this option and opt to participate in the study will return the necessary forms to the study centre. Randomisation will not take place since GPs voluntarily decide to participate in the QC or independent study (Fig. 1).

### Primary objective

This study seeks to establish whether or not the CME module “Medical rehabilitation provided by the German pension fund” improves the organizational practice of GPs as they prepare medical reports in support of applications for MR.

### Secondary objectives

- To assess how satisfied participants are with the CME module (QC versus independent study)
- To establish whether or not the CME module improves self-rated skills (QC versus independent study)
- To determine whether or not completion of the module leads to a gain in knowledge about rehabilitation (QC versus independent study)
- To investigate whether or not the module positively influences attitudes towards rehabilitation (QC versus independent study)
- To investigate whether or not the participation of the moderators in the optional informational meeting has an influence on the results

### Outcomes

- We focusing on self-rated skills (complete specific rehabilitative documents), knowledge (identification of patients with rehabilitation needs), attitudes (increasing of rehabilitation motivation of participants)
- The participants learn clinical decision making for patients with rehabilitation need.
- The approval rate of rehabilitation applications for which participating GPs have submitted medical reports during the 6 months before the intervention and 6 month after serves as the measure to assess the objective organizational practice necessary to prepare succesfully supporting medical reports.
- Measurement of participant satisfaction with the module will be done using adapted items from the HILVE-II questionnaire, [30] and open-ended questions.
- Perceived professional skills will be assessed using items from the BEvaKomp, [31].
- Knowledge of rehabilitative medicine will be assessed using validated, reviewed multiple-choice questions dealing with rehabilitative medicine, [32]
- Attitudes towards rehabilitation will be captured using items developed by the study authors.

### Intervention

The content of the CME module “Medical rehabilitation provided by the German pension fund” was developed based on the concrete need for information identified in earlier national studies, [8, 9]. The focus of the CME module is on reducing barriers of access to rehabilitation for general practitioners, recognizing the need for rehabilitation, and providing concrete guidance for preparing medical reports.

The educational strategy was developed over multiple phases in an interdisciplinary group of general practitioners, rehabilitative medicine specialists, and physicians who also function as QC moderators; consensus was reached with DRV representatives. A manual for moderators with pedagogical guidance, a slide presentation, and materials on case-based small-group assignments was created for holding the module as a series of on-site meetings; a folder for participants with concrete practical guidance was also developed. When designing this approach, particular value was placed on interactive and activating elements. At the end of the module, the rehabilitation-related information will be applied to two patient cases from general practice and discussed in depth. In addition, several moderated sessions will be held to address practical issues surrounding rehabilitation.

Completing the module as an independent study involves working through the contents of the participant folder with case-based exercises and a final exam based on validated, rehabilitation-related multiple-choice questions, [32].

Accreditation of the module has been granted by the medical board of Saxony-Anhalt (*Ärztekammer Sachsen-Anhalt*) with the assignment of five CME credits for participation in the module within the scope of an on-site general practice quality circle and three CME credits for independent study.

### Ethical considerations

This study was submitted to the ethics committee of the Martin-Luther-Universität Halle-Wittenberg for suggestions and approved under no. 2014–13. The study is listed in the German Clinical Trials Register (DRKS) under DRKS00006188 and on the WHO International Clinical Trials Registry Platform under Universal Trial Number (UTN): U1111–1158-8334.

### Study governance

The steering committee for this study includes the following authors: SF, KP, AW, WM and AK. The steering committee will be responsible for study design, start, evaluation and reporting the results of the trial. The steering committee will meet at least every 3 months throughout the study.

### Data management

Attendance records, consent forms and the questionnaires (t0, t1) for the QC participants will be compiled by the moderators and forwarded to the study centre. Those participating in the self-study option will be responsible for sending their consent forms, evaluation questionnaires and completed exams to the study centre.

At the study centre data will be compiled and kept by a custodian who is responsible for sending a list of the study participants to the DRV, where using billing data for each physician, the number of approved MR applications will be determined and reported to the study centre. The questionnaire for the follow-up survey (t2) will be sent out by the custodian. All data shall be compiled by the custodian using identification numbers and forwarded in pseudonymised form to the authors for analysis.

Data sharing is not applicable to this article. The datasets were analyzed during the current study.

### Publications

Our writing group will be formed by the steering committee and all authors will meet ICMJE criteria for authorship. Results will be disseminated in peer-reviewed journals and through presentation at national and international scientific meetings. Results will be communicated to the consumer advisory group and the study funders and collaborators.

## Discussion

We present an ambitious multi-level study protocol. We show an realizable intenvention concept of a difficult target group with a good response rate.

## Conclusion

Implementing targeted CME on complex topics such as those involving barriers is possible, even promising, when using QCs and their moderators. Of particular importance is how aware moderating physicians are of the relevance of MR need detection and access. We evaluate for the first time a CME module specifically designed to improve the rehabilitation-related expertise and competencies held by GPs in regard to a) subjective physician satisfaction with the CME module, b) stability of CME-based knowledge and attitudes over time, and c) changes in the objective and subjective MR-related competencies needed to support MR applications by comparing QC participants with the study participants who elected to cover the material individually.

### Study sponsorship

This study has received support from the Deutsche Rentenversicherung Bund; it is being undertaken with the support of and in collaboration with the Medical Faculty of the Martin-Luther-Universität Halle-Wittenberg, the Deutsche Rentenversicherung Mitteldeutschland (DRV), the Hausärzteverband Sachsen-Anhalt, and the Kassenärztliche Vereinigung Sachsen-Anhalt (KVSA).
